# Heterologous Expression of Recombinant Human Cytochrome P450 (CYP) in *Escherichia coli*: N-Terminal Modification, Expression, Isolation, Purification, and Reconstitution

**DOI:** 10.3390/biotech12010017

**Published:** 2023-02-07

**Authors:** Tao Shang, Chee Mun Fang, Chin Eng Ong, Yan Pan

**Affiliations:** 1 Division of Biomedical Science, School of Pharmacy, University of Nottingham Malaysia, Jalan Broga, Semenyih 43500, Malaysia; 2 School of Pharmacy, International Medical University, Bukit Jalil, Kuala Lumpur 57000, Malaysia

**Keywords:** human cytochrome P450, heterologous expression, *Escherichia coli*, purification, N-terminal modification

## Abstract

Cytochrome P450 (CYP) enzymes play important roles in metabolising endogenous and xenobiotic substances. Characterisations of human CYP proteins have been advanced with the rapid development of molecular technology that allows heterologous expression of human CYPs. Among several hosts, bacteria systems such as *Escherichia coli* (*E. coli*) have been widely used thanks to their ease of use, high level of protein yields, and affordable maintenance costs. However, the levels of expression in *E. coli* reported in the literature sometimes differ significantly. This paper aims to review several contributing factors, including N-terminal modifications, co-expression with a chaperon, selections of vectors and *E. coli* strains, bacteria culture and protein expression conditions, bacteria membrane preparations, CYP protein solubilizations, CYP protein purifications, and reconstitution of CYP catalytic systems. The common factors that would most likely lead to high expression of CYPs were identified and summarised. Nevertheless, each factor may still require careful evaluation for individual CYP isoforms to achieve a maximal expression level and catalytic activity. Recombinant *E. coli* systems have been evidenced as a useful tool in obtaining the ideal level of human CYP proteins, which ultimately allows for subsequent characterisations of structures and functions.

## 1. Introduction

Cytochrome P450 (CYP) enzymes are a group of membrane-bound hemoproteins responsible for the synthesis of a great number of endogenous compounds including steroid hormones, bile acids, fatty acids, and eicosanoids [1,2,3]. CYPs are also major phase I metabolizing enzymes, bio-transforming xenobiotics such as drugs and carcinogens, in the body [4,5]. In humans, the CYP families 1, 2, and 3 contribute significantly to xenobiotic metabolism, while other CYPs are mainly involved in endogenous biotransformation [6]. Unlike prokaryotic CYPs, which are soluble, mammalian CYPs are integral membrane proteins found in the endoplasmic reticulum or mitochondria [7]. Characterisations of the structure–function relationships for CYP enzymes have been impeded by the challenges of purifying these insoluble CYPs from human tissues with sufficient quantity and activity [8,9]. Moreover, with the advanced development of whole-genome sequencing technologies, a large number of CYP genomic variations have been identified [10]. CYP polymorphisms, in particular, CYP2C9, CYP2C19, and CYP2D6, account for the most commonly seen variations in phase I drug metabolism clinically [11]. Nevertheless, the low frequencies of CYP variants have limited the evaluations of their impact on the pharmacokinetics of clinical drugs [12].

The heterologous expression systems provide an alternative opportunity to obtain individual CYP isoforms and their variants in evaluating the enzyme activities or in analysing protein structures under reproducible conditions [13]. Thus far, several in vitro expression systems, including mammalian cells, baculoviruses, yeast, and bacteria cells, have been documented for applications in characterising CYP enzymes [14]. Mammalian cells such as the African green monkey kidney-derived cells COS-1 and the human embryonic kidney cells HEK293 have been employed in expressing recombinant human CYP enzymes [15,16]. The advantages of the mammalian cell systems include no requirement for cDNA modifications, as well as adequate levels of endogenous NADPH-CYP oxidoreductase (OxR) and cytochrome *b*_5_ to support electron transport and CYP catalytic activities [17]. However, employment of mammalian cells is often associated with high technical demand and a long duration of culture [18]. Besides, the CYP expression levels in mammalian cell cultures are usually low, which is unsuitable to study CYP variants, in particular, with low enzyme activity [14]. Baculovirus systems employ insect cells to express recombinant human CYPs, which can achieve high levels of expression [19]. Nevertheless, the technical demand and cost for insect cell cultures are high. The baculovirus systems also require the co-expression of OxR as insect cell lines are unable to express sufficient levels of OxR [17]. Yeasts such as *Saccharomyces cerevisiae* and *Schizosaccharomyces pombe* are useful in expressing human recombinant CYP [20,21]. The advantages of using yeast cells are low cost for maintenance, ease of culture, and a relatively high yield of CYP proteins. Moreover, the protein expression and post-translational modification processes are similar to those of higher eukaryotes, hence modifications of cDNA are usually not required [17]. Despite that yeasts contain endogenous OxR, the activity and quantity may be insufficient to fully support CYP enzyme activities, thus exogenous OxR may be essential [22]. Bacterial cells such as *Escherichia coli* (*E. coli*) demonstrate several advantages when being used as a heterologous system for human CYP expression. Culturing bacterial cells involves minimal maintenance cost as well as easier and faster cultivation. The recombinant CYP expression levels in bacteria are usually higher compared with those in yeast cells [23]. On the other hand, as human CYPs are membrane-bound, their expression in bacteria systems would require N-terminal modifications of the CYP cDNA to achieve optimal protein expression, conserve ideal folding, and maintain native biological functions [7,24].

Among these heterologous hosts for the purpose of expression of recombinant human CYPs, bacteria *E. coli* is the most commonly used. However, the levels of expression in *E. coli* reported in the literature sometimes differ significantly. Several contributing factors, including N-terminal modifications, co-expression with a chaperon, selection of vectors and *E. coli* strains, bacteria culture and protein expression conditions, bacteria membrane preparations, CYP protein solubilizations, CYP protein purifications, and reconstitution of CYP catalytic systems have been manipulated to allow maximal expression and purification of a multitude of human CYP proteins in bacterial systems [25,26,27]. Considering the number of variables responsible for optimal recombinant human CYP enzymes expressed in the bacteria systems, this paper explored and gathered successful recombinant expression designs to gain a collective understanding of maximal human microsomal CYP protein expression in bacteria cells.

## 2. Modifications of N-Terminus

**Figure 1:**
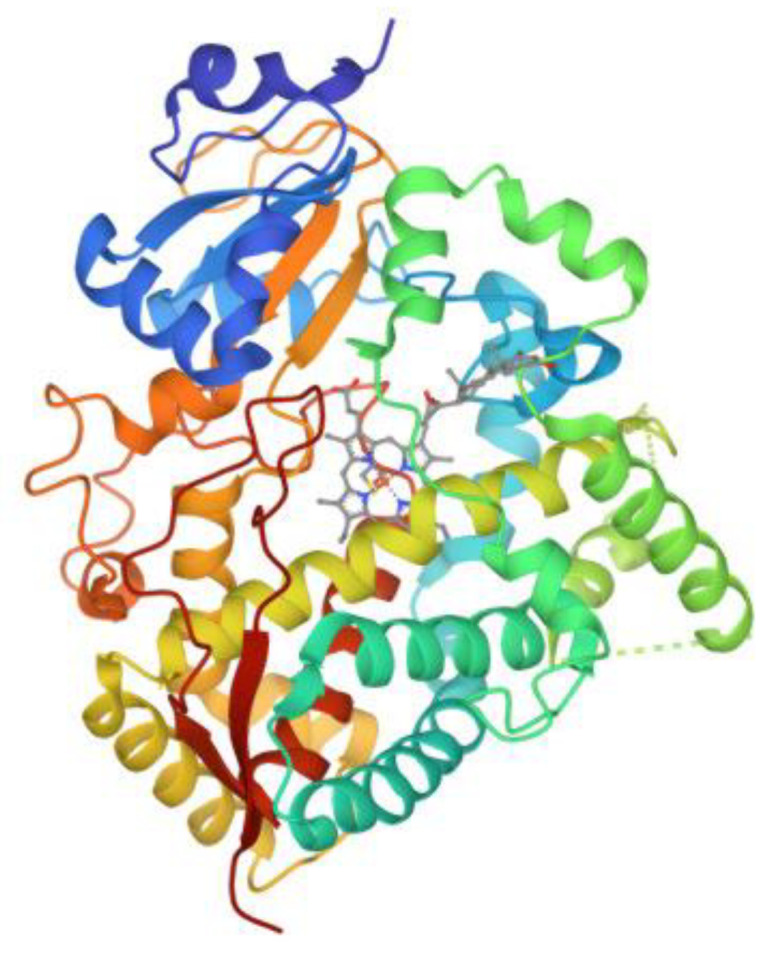
Crystal structure of human CYP3A4 (image from the RCSB PDB (rcsb.org) of 1W0F) [40].

In contrast to prokaryotic CYPs, mammalians including human CYPs are associated with membranes, making them insoluble. The rough endoplasmic reticulum (ER) and mitochondrial membranes are the major sites to which human CYPs are attached [28]. The CYP isoforms share around 40% sequence similarity with a common and highly conserved CYP fold [29]. It contains twelve α-helices (named A to L starting from the N-terminus) and a small percentage of β-sheets. The highly conserved I-helix plays an essential role in catalysis, while the F/G-loop, the F and G-helices, and the B/C-loop form a ‘lid’ over the active site cavity [29]. Figure 1 shows the structure of human CYP3A4 obtained by X-Ray diffraction as one example. CYPs can form dimers, trimers, and tetramers via multiple non-covalent interactions or covalent bonds, which is known as oligomerization. Conditions such as environmental pH, temperature, and the presence of lipids can affect their oligomerization states [30]. On the other hand, the oligomerization state can influence CYP enzymes’ kinetic properties and substrate specificities. Dimer formation resulted in enhanced catalytic efficiency of the CYP [31]. More details regarding the membrane effects on structure, ligand binding, as well as interactions with co-enzymes/co-factors can be found in a review by Martin and colleagues [32].

In humans, it is believed that the catalytic domain of CYPs and the N-terminus are located on the cytosolic side and luminal side of the ER, respectively [28,33]. The N-terminal transmembrane is a single α-helix containing a long stretch of hydrophobic amino acids, allowing this region to interact with the hydrophobic membrane environment on the ER [33]. Different CYP families consist of amino acids with a large variation on the N-terminal helix [34]. On the other hand, the mitochondrial CYPs have a topogenic sequence; hence, they do not need the N-terminal transmembrane anchor [35]. Prokaryotes and eukaryotes employ similar systems in the process of directing the protein to the membrane. However, a foreign signal peptide from the human CYPs may not be well-recognised by the bacterial expression systems, resulting in low levels of expression [36]. Insertion of *E. coli* leader sequences such as ompA and pelB into the beginning of the protein sequence has been led to the ability to obtain CYPs with a full length [37]. Alternatively, the alterations of CYP N-terminal membrane-directing signaling sequences lead to direct expression of CYP at the plasma membrane because bacteria cells have no organelles [23]. The common N-terminal modifications employed for human CYP expression in bacterial systems include truncations of the N-terminus, incorporation of the ‘LLLAVFL’ sequence, substitutions of N-terminal sequence with hydrophilic residues, and silent mutations to optimise AT content [7,38,39]. Zelasko and coworkers performed a thorough review of how these N-terminal modifications were applied in optimising recombinant CYP yields in *E. coli* [23].

### 2.1. N-Terminal Trucation

Partial or complete truncation of the N-terminal anchor sequence of the CYP protein would likely increase its solubility in the cytoplasm [41]. It is difficult to attain fully soluble CYP protein only by modifying the N-terminal sequence, as other parts of the CYP protein such as F-G helices are also responsible for the membrane anchor [30]. The removal of the N-terminal sequence has been applied to various human CYP expressions in bacteria systems such as CYP2E1, CYP3A4, CYP2B4, CYP1A1, CYP1A2, CYP2D6, CYP1B1, CYP2A6, and CYP2S1 [7,38,39,42,43,44,45,46,47,48]. However, the expression yields and enzyme activities varied dramatically from one study to another. Human CYP2E1 lacking residues 3–29 demonstrated comparable catalytic activity to the native protein, suggesting that this segment has no function in its oxidative activity [7,49]. Some enhancement of human CYP3A4 expression yield was observed in the construct with the removal of residues 3–24 [42]. However, truncation of CYP2B6 cDNA did not enhance protein expression yield in bacterial cells [47].

### 2.2. Substitutions of N-Terminal Sequences with Hydrophilic Residues

Apart from the truncation of the N-terminal sequence to improve the overall hydrophilicity of human CYP proteins expressed from bacteria cells, additional hydrophilic or charged sequences such as ‘AKKTSS’, have been inserted at the N-terminus [31]. The incorporation of ‘AKKTSS’ is likely to aid in the solubilization of several human CYP enzymes including CYP2C8, CYP2C9, CYP2A6, and CYP4X1 [31,50,51,52]. Solubilization of CYP protein is essential for the subsequent crystallisation processes in order to obtain structural information. Collectively, N-terminal truncations or insertions of hydrophilic residues primarily affect CYP protein localisation between cytosol or membrane, and they alone seem to not always correlate with the enhancement in expression. Other modifications within the N-terminal region should be considered.

### 2.3. Incorporation of the ‘LLLAVFL’ Sequence

Barnes and colleagues introduced residues ‘LLLAVFL’ at the N-terminus of bovine CYP17α hydroxylase protein to produce optimal protein expression and activity [25]. This N-terminal ‘LLLAVFL’ is a binding consensus sequence of the ribosome, which enables maximal ribosome recognition and translation initiation across many human CYP isoforms including CYP1A2, CYP2B6, CYP2D6, CYP3A4, CYP2C19, and CYP3A43 [38,47,53,54,55]. Nevertheless, Sandhu and coworkers reported that constructs containing this sequence did not yield any spectrally detectable CYP2C9 [24].

### 2.4. Silent Mutations

In *E. coli*, the translation of mRNA codons to amino acids involves the ribosome containing a 30S and a 50S subunit. The translation process starts with the recognition of the start codon (AUG) and subsequent binding of the 30S ribosomal subunit to the Shine–Dalgarno sequence AGGAGG. Any form of secondary or tertiary structures may block this ribosomal binding [56]. Optimisations of nucleotides in this region to avoid secondary structures have been shown to enhance protein expression by increasing ribosomal binding. Therefore, a silent mutation that does not change the protein’s amino acid sequence has become one of the strategies in the heterologous expression of human CYPs in bacterial systems. However, several studies found that silent mutations alone were not usually sufficient for maximal expression, and concurrent modifications of the N-terminus were often required [38,39]. The silent mutations often involved the enhancement of AT content over the first few codons, which minimised the potential of mRNA secondary structure formation by reducing the free energy [45,46].

Moreover, *E. coli* ribosomes are not able to recognise and bind some eukaryotic codon sequences because bacterial cells may lack the corresponding tRNA. This codon bias showed a significant correlation with transcription efficiency at the N-terminus [57]. Apart from minimising the mRNA secondary structure, favouring *E. coli* codons through silent mutation may also facilitate heterologous CYP protein expression. According to a review, *E. coli* preferred to translate certain codons that are different from those of humans. For instance, *E. coli* frequently employs CUG for coding leucine, GGU for glycine, and AAA for lysine [58]. Several studies have employed in silico tools such as DNAWORKS from the National Institutes of Health to incorporate automatic codon optimisation to fulfil the codon preference bias of *E. coli* such as CYP2W1, CYP4X1, and CYP2S1 [48,52,59,60].

### 2.5. Use Codons Encoding Alanine as the Second Codon

It is known that the presence of mRNA secondary structure in the binding sites of the ribosome potentially inhibits the gene expression. In addition to the silent mutations described above to minimise the secondary structure, mutation of the second codon to alanine has been shown as an effective approach to maximise protein expression in bacterial systems [61]. Following Barnes et al.’s successful CYP17α expression optimisation by mutating the second codon to alanine, the majority of the subsequent heterologous expression of human CYP in bacteria cells incorporated the alteration of the second codon to code for alanine [25]. Many of them demonstrated enhanced protein expression in the testing systems [44]. Nevertheless, similar to other modifications, alteration of the second codon alone has been insufficient, but additional N-terminal changes were required to achieve optimal expression [39,45].

## 3. Co-Expression with Chaperon

*E. coli* usually degrades misfolded proteins rapidly [62]. A chaperon system can facilitate the correct folding and proper incorporation of heme into CYP protein by supplying a hydrophilic environment [63]. Thus, CYP expression yields can be increased dramatically. Co-expression of recombinant human CYPs with molecular chaperon GroES-GroEL has been frequently employed. Many researchers have adopted this system with higher levels of protein expression, including CYP1A2, CYP2W1, CYP2B6, CYP4X1, and CYP2J2 [9,26,52,59,64].

## 4. Selections of Expression Vectors and *E. coli* Strains

The successful expression of CYP protein in bacteria is also influenced by the choice of plasmid vectors and *E. coli* strains (see Table 1).

The most commonly employed CYP expression plasmid vector in *E. coli* is pCWori+. It was initially developed by F.W. Dahlquist and is not commercially available [23]. The overall structure of pCWori+ has been illustrated previously [65]. Essentially, it contains two tac promoters upstream of the Nde I restriction enzyme digestion site coincident with the ATG codon (start codon). Only one tac promoter (the one upstream of the polylinker site) is used, which is recognised by *E. coli* RNA polymerase. Upon the addition of Isopropyl β-D-1-thiogalactopyranoside (IPTG), the protein expression output is proportional to the amount of IPTG, which allows the expression of the precise level of CYP [23]. Additionally, it contains one trpA (a strong transcription terminator), the β-lactamase gene (conferring ampicillin resistance), and the lacI^q^ gene that encodes the Lac repressor (prevents any transcription initiated from the tac promoters without adding inducing agents) [65]. In general, the target CYP cDNA (native or modified) is introduced between the ATG start codon (contained within the Nde I site) and another restriction enzyme site, which is usually carried out by polymerase chain reaction (PCR) mutagenesis [25].

The recombinant vector was used in the transformation of various *E. coli* strains to produce recombinant human CYP proteins. Among them, DH5α [8,9,24,26,27,38,39,42,44,45,46,52,53,59,66,67,68,69,70,71,72,73,74,75,76] and JM109 [24,25,38,64,77,78,79,80,81] strains are the most commonly used, while MV1304 [7,43,47,49], XL-1 blue [82], and TOPP [83,84] have also been used. It is important to note that the *E. coli* strain selection can impact CYP expression levels. It was evidenced that CYP2C10 was not detectable in JM109 cells, but expressed in DH5α cells [24]. Nevertheless, no genetic markers were identified in these strains, showing a significant correlation with the capability of producing high levels of recombinant CYP proteins [65]. It is suggested to evaluate these common *E. coli* strains for their ability to express a particular recombinant CYP at the beginning of the study.

**Table 1:** External contributing factors for selected human CYP expression in *E. coli*.

CYP	Expression Vector	*E. coli* Strain	LB to TB Ratio	OD_600_	Temp (°C)	Shaking Speed (rpm)	Duration (hour)	IPTG (mM)	Δ-ALA (mM)	Reference
2E1	pCWori+	MV1304	1:90	0.5–0.8	N/A	N/A	4	1	N/A	[7]
17A1	pCWori+	JM109	N/A	0.4–0.8	28	N/A	48	1	N/A	[25]
1A2	pCWori+	DH5α	1:100	N/A	30	125	72	1	N/A	[66]
3A4	pCWori+	DH5α	1:100	N/A	32	N/A	24	1	N/A	[42]
2E1&2B4	pJL	MV1304	N/A	1.0	N/A	N/A	4	1	N/A	[43]
1A1	pCWori+	DH5α	1:100	N/A	30	Vigorous	48	N/A	N/A	[44]
2E1	pCWori+	DH5α	1:100	N/A	30	Vigorous	48	1	N/A	[68]
1A2	pCWori+	DH5α or JM109	1:100	N/A	30	Vigorous	48	1	N/A	[38]
7A1	pJL	TOPP3	N/A	0.4–0.6	30	150	15–18	1	0.2	[83]
17A1-OxR	pCWori+	DH5α	1:111	N/A	27	125	72	1	N/A	[69]
3A5	pCWori+	DH5α	1:100	N/A	30	220	24	1	N/A	[70]
2D6	pDS9	JM109	1:10 to 40	0.7–0.9	23	100	48	5 µM	0.5–1	[77]
2D6	pCWori+	DH5α	N/A	N/A	30	200	43	1.5	0.5	[39]
2E1-OxR	pJL2	XL Blue	N/A	0.8	26	100	60	1	N/A	[82]
1A1-OxR	pCWori+	DH5α	1:100	N/A	28	125	48	1	0.5	[71]
27A1	pTrc99A	TOPP3	1:100	N/A	29	210	48	0.5	0.5	[84]
1A2-OxR	pCWori+	DH5α	1:100	N/A	28	125	48	1	0.5	[85]
1B1-OxR	pCWori+	DH5α	1:100	N/A	30	200	24	1	0.5	[45]
3A5	pCWori+	DH5α	1:100	0.3	30	160	28	0.1	1	[72]
2A6	pCWori+	DH5α	1:100	N/A	32	200	40	1	0.5	[46]
2B6	pCWori+	MV1304	N/A	N/A	28	200	40–48	1	0.5	[47]
2D6-OxR	pCWori+	DH5α	1:100	0.6–1.0	26	190	40–48	1	0.5	[27]
1A2-HDJ-1	pCWori+	DH5α	N/A	0.4–0.5	37	N/A	24	1	1.5	[26]
2B6-GroES/EL	pCWori+	JM109	1:100	N/A	30	160	72	1	0.5	[64]
27C1	pCWori+	JM109	1:100	N/A	27	200	48	1	0.5	[79]
4X1	pCWori+	DH5α	1:100	0.5	28	190	17–21	1	0.5	[52]
2S1	pBdtacHR	LMG194	1:400	N/A	30	115	24–36	0.5	0.5	[48]
1A1-OxR	pCWori+	DH5α	1:1000	0.5–0.7	30	200	24	1	0.5	[8]
2C10 &2C9	pCWori+	DH5α and JM109	1:100	N/A	30	Vigorous	24	1	N/A	[24]
4A11	pCWori+	DH5α	N/A	0.5	28	200	48	1	0.5	[74]
2J2	pCWori+	DH5α	N/A	N/A	28	N/A	48	1	0.5	[75]
4B1	pCWori+	DH5α	N/A	0.4	27	120	48	1	0.5	[76]
39A1-GroEL/ES	pCW-LIC	C41	N/A	0.6	26	110	48	0.5	0.5	[86]
2J2-GroEL/ES	pCWori+	DH5α	1:100	0.4–0.6	28	180	48	1	0.5	[9]

N/A = not available.

## 5. Bacteria Culture and Protein Expression Conditions

The typical bacteria culture and protein expression start with the initial culture of transformed *E. coli* strain in LB media supplemented with ampicillin (50–100 µg/mL) overnight at 37 °C (the optimal growth temperature for *E. coli*), followed with growing in Terrific Broth (TB) media containing ampicillin for an extended number of hours. The protein expression is subsequently induced by adding an inducing agent such as IPTG [42]. Factors involved in this process that may affect the yield of CYP protein expression include the ratio of LB to TB, OD_600_ readings upon initiation of protein expression, temperature, shaking speed, expression duration, concentrations of IPTG, with or without δ-aminolevulinic acid (δ-ALA), and other more specific conditions for a particular CYP isoform (see Table 1).

TB is a type of phosphate-buffered media that maintains a neutral pH level and comprises readily utilisable carbon sources [65]. The LB culture-to-TB culture ratio is usually maintained at 1:100 (e.g., 10 mL of LB culture to 1 L of TB) [42,66]. The TB media is often supplemented with trace elements to maintain CYP enzyme stability. Different studies applied different trace element compositions. As reported by Ahn and colleagues, trace elements expressing CYP1A2 in *E. coli* included 50 µM FeCl_3_, 1 mM MgCl_2_, and 2.5 mM (NH_4_)_2_SO_4_ [26]. It is common for 1 mM thiamine (also known as vitamin B1) to be added to the TB culture media to ensure rapid *E. coli* growth [87]. The typical OD_600_ values of 0.4 to 0.8 representing the mid-exponential bacterial growth phase were mostly used prior to induction [7,27]. Arabinose was required to induce the chaperon GroES-GroEL [9,48,52].

IPTG is a compound that mimics the molecular structure of allolactose that triggers the transcription of *lac* operon in *E. coli*. Hence, IPTG is used for protein expression induction where the gene expression is controlled by the *lac* operator, including pCWori+, the most commonly used vector for heterologous CYP protein expression in *E. coli* [65]. The majority of the studies employed 1 mM IPTG to induce CYP expression in *E. coli* cells, while exceptions were found in the expressions of CYP2D6 (1.5 mM IPTG) [39], CYP3A5 (0.1 mM IPTG) [72], CYP2S1, and CYP39A1 (0.5 mM) [48,86]. Δ-ALA, a well-known heme precursor, is involved in the pathway of protoporphyrin IX synthesis, and thus heme synthesis [88]. *E. coli* cells are able to produce heme-containing proteins with their endogenous heme biosynthesis system. The current results show that, although not an exclusive requirement for maximal production of all human CYP proteins in *E. coli*, the supplementation of δ-ALA could enhance the expression dramatically [65]. δ-ALA is readily taken up by *E. coli* cells, followed by heme synthesis catalysed by bacterial enzymes, which is subsequently inserted into the recombinant CYP polypeptide to form an enzymatically active protein [89]. The most commonly used final concentration of δ-ALA added before induction is 0.5 mM, with exceptions such as 1 mM for CYP3A5 [72] and 1.5 mM for CYP1A2 [26]. The addition of other chemicals to expression media was more specific to one or a group of CYP proteins. 4-methyl pyrazole, an inhibitor of CYP2E1 with high affinity, was added to the expression culture to stabilise the protein [7,82,90]. Bactopeptone was seeded in a TB medium to enhance cell growth in several studies [24,26,47,71].

The employment of 37 °C for protein expression usually results in recombinant CYP accumulating as inclusion bodies. A lower expression temperature has been shown to produce more stable proteins without aggregation [91]. Nevertheless, expression temperatures below 25 °C lead to a dramatic drop in the expression level [23]. The optimal expression temperature during protein induction is often within a rather narrow range, and thus sensitive to drastic fluctuations in the temperature of the incubator. The typical induction temperature is not higher than 30 °C (mostly 28–30 °C). Certain human CYP proteins can be expressed with higher yields under higher temperatures, such as CYP2A6, CYP2E1, and CYP1A2, which were expressed at a comparable level and activities at 37 °C [26,92]. Moreover, the shaking speed and length of incubation during induction may also influence the optimal expression levels. The culture media in flasks shaken vigorously at 100–200 rpm were routinely performed to obtain optimal yields [47,76]. During the induction phase, the incubation usually lasts for 24–72 h. For instance, Bui and Hankinson reported that the growth of *E. coli* at 30 °C for 24 h provided the best expression conditions for a recombinant CYP2S1 [48].

## 6. Membrane Isolation

At the end of protein expression, bacterial cells are harvested by centrifugation, followed by membrane isolation prior to purification. The general steps of membrane isolation include suspension of harvested cells, lysis of cells, removal of cell debris, and membrane fraction sedimentation by ultra-centrifugation. Different studies applied different protocols in terms of suspension buffer, lysis of cell methods (by a high-pressure homogenizer, lysozyme, and ultrasonic energy), choice of a protease inhibitor, and collection of membrane fraction sedimentation.

The harvested cells were usually suspended in phosphate buffers [49,86] or tris acetate buffers [38,42] with a pH range of 7.4–7.8 containing additional common compositions such as ethylenediamine tetraacetic acid (EDTA), sucrose, dithiothreitol (DTT), and glycerol. All of the steps were carried out at 4 °C. Both buffers functioned equally well in suspending bacterial cells expressing various recombinant human CYP proteins. Bacteria cells were suspended in a concentrated sucrose solution supplemented with EDTA, which were subsequently re-suspended in cold water. Under this condition, the bacteria cells shrink as a result of the high osmotic strength of the sucrose solution. EDTA plays a role in releasing lipopolysaccharide (LPS) from the cell envelope of bacterial cells, hence increasing the permeability of the outer membrane. Cold water leads to the rapid enlargement of cell size, resulting in the release of periplasmic proteins. This technique for the recovery of recombinant protein from *E. coli* is known as an osmotic shock [93]. Serious challenges have occurred in preserving protein stability and activity in biological applications as they are just marginally stable [94]. DTT is one of the protein reductants responsible for breaking down protein disulfide bridges and stabilizing enzymes [95]. Moreover, the most widely employed co-solvents for protein stabilization are polyols and, among polyols, glycerol is one of the most commonly used to stabilize and avoid aggregation of the protein [96,97].

Cell lysis can be defined as the destruction of the outer boundary or cell membrane to release inter-cellular materials. Cell lysis methods can be classified into mechanical (such as high-pressure homogenizer and bead mill) and non-mechanical approaches (including physical and chemical disruption) [98]. For the lysis of *E. coli* cells to obtain expressed human CYP proteins, mechanical approaches that use high-pressure homogenizer and non-mechanical techniques employing ultrasonic cavitation and enzymatic cell lysis were often recorded. A high-pressure homogenizer disrupts the membrane of cells by forcing them through an orifice valve [7,86]. Additionally, lysozyme is usually added to the suspended cell solution and incubated on ice or at 4 °C with stirring or shaking for 30 min [8,53]. Lysozyme is specific towards bacterial cells and reacts with the peptidoglycan layer, leading to the breaking of the glycosidic bond in the bacterial cell wall [99]. Ultrasonic cavitation is routinely applied in laboratories to disrupt cells. Ultrasound waves generate ultrasonic energy, which is transferred into the liquid solution and results in negative pressure. Once the negative pressure is lower than the vapour pressure of the liquid, vapour-filled bubbles are formed in the liquid solution. Then, when the bubbles grow to the size at which the ultrasonic energy is insufficient to maintain the vapour inside, they collapse and release a large amount of mechanical energy in the form of a shock wave, leading to cell rupture [100]. One of the disadvantages of ultrasonic cavitation is the generation of a large amount of heat, which may degrade enzymes [98]. During the lysis of *E. coli,* cells to isolate recombinant CYP proteins, a few rounds of ultrasonic treatment along with intervals on the ice were carried out in an ice bath to maintain cold conditions [24,25].

Upon lysis of cells, proteases are also released and their digestive functions are triggered, which can degrade isolated CYP enzyme proteins. Hence, the addition of protease inhibitors is required to preserve protein from imminent natural degradation. The majority of the proteases found in *E. coli* cells belong to the class of the serine protease group. Among the many classes of protease inhibitors, phenylmethylsulfonyl fluoride (PMSF) that inhibits serine protease irreversibly by deactivating the serine hydroxyl group is the most commonly used [101]. More recently, protease inhibitor cocktails comprising a mixture of several inhibitor compounds are more preferred in targeting a wide range of proteases that degrade enzymes via different mechanisms [27,81].

It was demonstrated that recombinant CYP proteins were anchored to the inner membrane of *E. coli* cells [49,68]. Ultracentrifugation with a speed of 100,000–225,000 g for a duration of 30–180 min was carried out to separate the membrane protein fraction (containing CYP) from other cytoplasmic soluble proteins and the majority of nucleic acids. The conditions described above are summarised in Table 2.

## 7. CYP Protein Solubilization and Purification

As noted above, isolated CYP proteins are bound to bacteria membranes, and solubilization with appropriate detergents is essential prior to protein purification. The desirable properties of the detergents used for this purpose include the following: (i) good solubilizing power; (ii) low tendency towards protein denaturation; (iii) can be removed by dialysis or dilution easily; (iv) optical transparency to allow detection of protein using a spectrophotometer; (v) free of interference with protein determinations; (vi) owning non-ionic properties for ion-exchange chromatography and isoelectric focusing; (vii) good solubility; (viii) simple procedure of detergent determination; (ix) stable; and (x) affordable cost [102].

Among these detergents, *n-ocylglucoside*, also known as octyl β-D-glucopyranoside, is a non-ionic surfactant endowed with a majority of the desirable properties listed above; however, it is rather expensive, limiting its application mostly to small-scale experiments [103]. It has been applied to solubilize CYP2E1, CYP1A2, and CYP3A4 [7,49,104]. Sodium cholate is a type of bile acid salt that is a biologically active anionic detergent [105]. It consists of a hydrophobic steroid nucleus, three hydroxyl groups, and one ionic head of a carboxyl group [106]. Sodium cholate was one of the most employed detergents in the literature for solubilizing CYP proteins from *E. coli*, which was used alone [42,84] or more commonly used together with another non-ionic detergent such as Triton N-101 [38,44,45,46,47,64,68,70,73], or less often with Tergito NP10 [59,79] and Emulgen 911 [8,24]. The combination of ionic (e.g., sodium cholate) and non-ionic detergents (e.g., Triton N-101, Tergitol NP-10, or Emulgen 911) has been more effective for the solubilization of some CYP proteins [107,108]. Besides, Emulgen 911 or 913 was also often employed as a single detergent in this process [27,67,69,71,84,85]. Another commonly used detergent to enhance CYP solubilization is CHAPS, which is a non-denaturing zwitterionic detergent [9,48,52,59,74,75,81]. Several other detergents were also seen during CYP solubilization from *E. coli* membranes including Triton X-114 [39], C_12_E_9_ [77], Nonidet-P40 [78], and Renex-690 [72].

Detergents that remained in the purified enzyme samples potentially modulate enzymatic activity [107]. Non-ionic detergents generally produce more inhibition than either zwitterionic or ionic detergents [109]. Moreover, detergents including Tritons X100 and X114, Emulgens 911 and 913, and Tergitol NP-10 were seen to be oxidized by CYP enzymes [110]. Once the isolated *E. coli* membrane containing CYP proteins is solubilized, the membrane solution is subject to various columns for chromatography purification to obtain CYP proteins and remove detergents. The commonly used chromatography methods include anion-exchange chromatography (in particular, diethylaminoethyl (DEAE)), cation-exchange chromatography (in particular, carboxymethyl (CM)), and hydroxylapatite chromatography in the presence of non-ionic detergent [111]. Ion-exchange chromatography is used to separate proteins and other components according to their net charge. Proteins with negative charges (anionic proteins) can be purified by chromatography of positively charged DEAE-cellulose and proteins that are positively charged (cationic proteins) can be purified with negatively charged CM-cellulose columns [112]. Typically, many membrane proteins of *E. coli* solubilized using sodium cholate and Triton N-101 were found to be bound to the DEAE-Sephacel column and the recombinant human CYP protein was eluted in the void volume. The remaining proteins with a low molecular weight could be subsequently removed by adsorption to a CM-Sepharose Fast-Flow column. Finally, detergents were removed by dialysis and adsorption to the hydroylapatite column [68].

Besides, the addition of His residues at the N- or C-terminus has been performed to facilitate protein purification [113]. These, added free His residues, are able to chelate Ni^2+^, hence application of the Ni^2+^-chelate affinity column allows rapid purification. Such strategies have been used with CYPs, with most of the His tags at the C-terminus [8,47,48,52,59,72,75,79,81] or, to a lesser degree, at the N-terminus [77,86]. Compared with the traditional ion-exchanged chromatography approaches described earlier, metal affinity methods have advantages such as (1) reducing the use of non-ionic detergents that are difficult to remove and can be inhibitors or substrates of CYPs [110] and (2) the studies of CYP mutants sometimes require a more rapid purification process as mutants are relatively less stable [114]. Table 3 provides examples of detergents and columns employed for human CYPs expressed in *E. coli* cells.

## 8. Reconstitution of CYP Enzyme Assay Systems In Vitro

Reactions catalysed by human CYP enzymes involve two electron transferences from the redox partner. NADPH-CYP reductase (OxR) functions as the redox partner, transferring both electrons required for the catalytic cycle. Some CYP reactions employ cytochrome *b*_5_ to transfer the second electron [115]. Successfully purified CYP proteins are usually characterised by their functions, structures, and interactions with other proteins. In this review, we focus on the reconstitution of CYP enzyme assay conditions in vitro by revealing how factors such as sources of OxR, presence of cytochrome *b*_5_, the ratio of OxR to CYP, and lipid compositions affect CYP catalytic properties.

OxRs from different sources including purified rat [9,25,84,86] or rabbit [24,38,39,42,44,45,46,49,68,70,71,73,77,85] liver microsomes, recombinant OxR [9,26,27,47,66,74,75,81,104], co-expression with CYP [48,52,53,67,80,82], and commercial products [64,72,78,83] were employed by different studies (see Table 3). Although immunological differences were observed among OxRs isolated from rats, rabbits, and human liver microsomes, the OxRs prepared from the three species were all able to reduce CYPs at relatively similar rates [116]. Besides, molecular techniques have been developed to obtain recombinant OxR proteins or to co-express OxR and CYP proteins from *E. coli* at the same time. In order to achieve the co-expression of OxR and CYP, several strategies have been applied, including (1) co-expression as a fusion protein [67]; (2) expression of both CYP and OxR from one plasmid [48,52,80,82]; and (3) expression of CYP and OxR from two independent plasmids [53,54,117,118]. The ratio between OxR and CYP could affect the CYP-catalysed reaction kinetics. It was seen that the CYP1A2 and CYP2A6 catalytic activities began to saturate when the OxR was twice (2:1 molar ratio) that of these two CYPs [119]. In liver microsomes, the concentration of CYP protein is significantly higher than the level of OxR (20:1) [120]. Under this condition, a single OxR molecule must transfer electrons to a number of CYP proteins, and it requires a highly organised system to regulate substrate metabolism effectively. The lipid bilayers of the membrane would provide facilitation to assemble such a system [115]. Together with their redox partners, human CYP enzymes are mainly embedded in the endoplasmic reticulum membrane and phospholipids are essential for their catalytic reactions [121]. In vitro reconstitution systems for CYP activities have employed lipids such as dilauroylphosphatidylcholine, phosphatidycholine, phosphatidylethanolamine, phosphatidylserine, and phosphatidic acid [122,123,124,125]. Different lipid compositions in the reconstitution systems influenced the rate of substrate metabolism, incorporation of CYP into the membrane, and enzyme stability [115,126,127,128,129]. As mentioned above, the second electron required for the reduction of CYP in the reaction cycle can be supplied by cytochrome *b*_5_ as well. Additionally, cytochrome *b*_5_ plays other vital roles in the monooxygenase system [130,131]. Cytochrome *b*_5_ was also able to modulate the activities of several CYP enzymes [132,133]. Many reconstitution enzyme systems for recombinant CYP expressed from *E. coli* included cytochrome *b*_5_ coupled with OxR [24,38,39,42,44,45,68,70,71,72,78,82,86], but it did not enhance the CYP39A1-catalysed reaction [86] (see Table 3).

## 9. Conclusions

**Figure 2:**
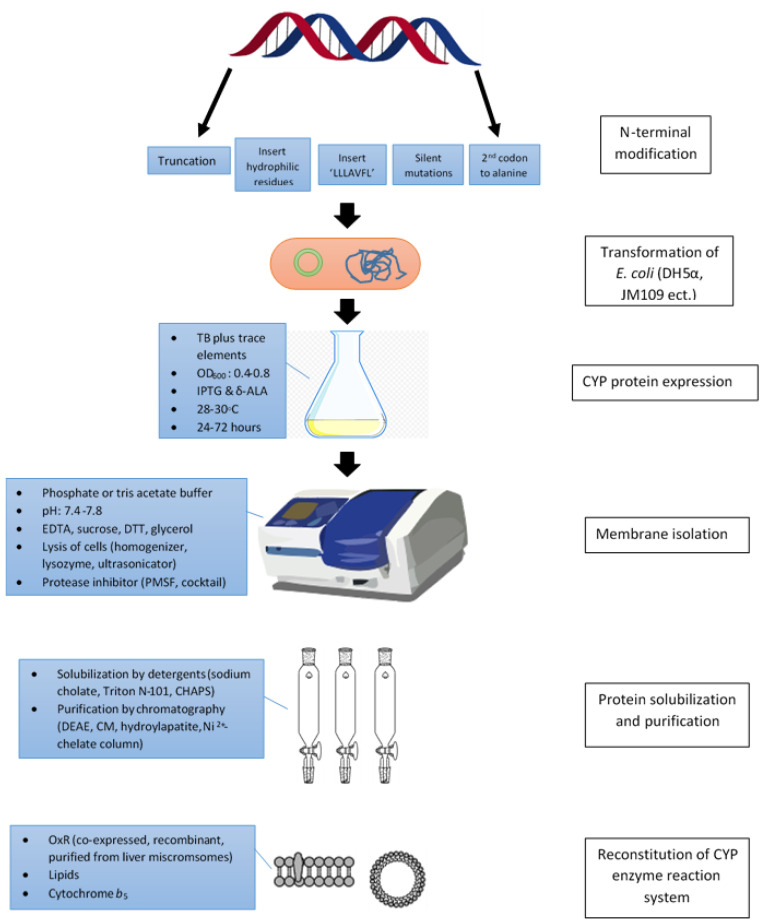
An overview of factors that determine successful recombinant human CYP expression from *E. coli* cells.

The factors affecting the successful expression and reconstitution of recombinant human CYP from *E. coli* in vitro include N-terminal modifications of CYP cDNAs, co-expression with a chaperon, selection of expression vectors and *E. coli* strains, bacteria culture and protein expression conditions, membrane and isolation conditions, CYP protein solubilization and purification, and in vitro reconstitution of CYP enzyme assay systems. Figure 2 provides an overview of these factors. It was observed from the collected findings that some alterations might not produce active CYP enzymes. Hence, each factor should be evaluated carefully to establish a system with high efficiency for a particular CYP isoform. In general, N-terminal modifications are essential to improve CYP solubilization status by truncation of the hydrophobic N-terminal region, the addition of 17α sequence (LLLAVFL), silent mutations to reduce secondary mRNA structure, and the substitution of the second codon to alanine. Additionally, co-expression with protein GroES-GroEL chaperone can facilitate proper CYP folding. pCWori+ vector is the most popular expression plasmid used for cloning recombinant CYP and to transform several *E. coli* strains such as DH5α and JM109. External bacteria culture and protein expression conditions such as OD_600_ readings upon initiation of protein expression, temperature, shaking speed, expression duration, and concentrations of IPTG, with or without δ-ALA, have the potential to significantly influence the expression yields. With regard to the membrane isolation, choices of suspension buffer, lysis of cell methods (by a high-pressure homogenizer, lysozyme, and ultrasonic energy), and the choice of protease inhibitor can be optimised to improve CYP protein yields. Various types of detergents (most often non-ionic plus ionic) were employed to solubilize expressed CYP proteins, followed by purification through ion-exchange chromatography. His tags can be attached to the C or N terminal of CYP cDNA for easier purification through affinity chromatography. Reconstitution of CYP reactions involves the construction of conditions similar to the native environment by including redox partners such as OxR and cytochrome *b*_5_ together at a suitable ratio with the appropriate type and level of lipids.

Recombinant *E. coli* systems have been evidenced as a useful tool in obtaining the ideal level of expression of human CYP proteins, which ultimately allows for subsequent characterisations of structure and functions. Moreover, it was noticed that the majority of the studies were reported before 2015. Hence, there is a need to develop and employ novel technologies for CYP protein expression and purification from bacterial cells.

## Figures and Tables

**Table 2:** Conditions of *E. coli* membrane isolations for selected human CYPs.

CYP	Suspension Buffer	Lysis Approach (yes/no)			Protease Inhibitor	Ultra Centrifugation	Reference
		High-Pressure Homogenizer	Lysozyme	Ultrasonic Cavitation			
2E1	Potassium phosphate buffer	Yes	No	No	N/A	142,000 g for 1 h	[7]
17A1	Mops ** buffer	No	Yes	Yes	PMSF, leupetin, aprotinin	225,000 g for 30 min	[25]
1A2	Potassium phosphate buffer	Yes	No	No	N/A	100,000 g for 60 min	[66]
3A4	Tris acetate buffer	No	Yes	Yes	PMSF, leupeptin, aprotinin, bestatin	180,000 g for 65 min	[42]
1A1	Tris acetate buffer	No	Yes	Yes	PMSF, leupeptin, aprotinin, bestatin	180,000 g for 65 min	[44]
2E1	Tris acetate buffer	No	Yes	Yes	PMSF, leupeptin, aprotinin, bestatin	180,000 g for 65 min	[68]
1A2	Tris acetate buffer	No	Yes	Yes	PMSF, leupeptin, aprotinin	180,000 g for 65 min	[38]
7A1	Potassium phosphate buffer	No	Yes	No	PMSF	100,000 g for 60 min	[83]
17A1-OxR	Tris-HCl buffer	No	Yes	Yes	PMSF	100,000 g for 60 min	[69]
2D6	Potassium phosphate buffer	Yes	No	No	PMSF, leupeptin	142,000 g for 60 min	[77]
2D6	Tris acetate buffer	No	Yes	Yes	PMSF, leupeptin, aprotinin, bestatin	100,000 g for 45 min, supernatants further centrifuge at 100,000 g for 16 h	[39]
2E1-OxR	Potassium phosphate buffer	Yes	No	No	N/A	142,000 g for 1 h	[82]
1A1-OxR	Tris acetate buffer	No	Yes	Yes	PMSF, leupeptin, aprotinin, bestatin	180,000 g for 65 min	[71]
27A1	Potassium phosphate buffer	No	Yes	Yes	PMSF	146,000 g for 60 min	[84]
3A5	Tris acetate buffer	No	Yes	No	PMSF, aprotinin	193,000 g for 40 min	[72]
2A6	Tris acetate buffer	No	Yes	Yes	PMSF, leupeptin, aprotinin, bestatin	180,000 g for 65 min	[46]
2B6	Tris acetate buffer	No	Yes	Yes	PMSF, leupeptin, aprotinin	180,000 g for 65 min	[47]
2D6-OxR	Tris acetate buffer	No	Yes	Yes	PMSF, protease inhibitor cocktail	100,000 g	[27]
1A2-HDJ-1	Tris acetate buffer	No	Yes	Yes	PMSF, leupeptin, aprotinin	180,000 g for 65 min	[26]
2B6-GroES/EL	Tris-HCl buffer	No	Yes	Yes	N/A	100,000 g for 60 min	[64]
27C1	Tris acetate buffer	No	Yes	Yes	PMSF, leupeptin, aprotinin, bestatin	180,000 g for 65 min	[79]
4X1	Tris acetate buffer	No	Yes	Yes	PMSF, leupeptin, aprotinin, bestatin	180,000 g for 65 min	[52]
2S1	Potassium phosphate buffer	Yes	No	No	PMSF	N/A	[48]
1A1-OxR	Tris acetate buffer	No	Yes	Yes	N/A	100,000 g for 75 min	[8]
2C10 &2C9	Tris acetate buffer	No	Yes	Yes	PMSF, leupeptin	180,000 g for 65 min	[24]
2J2	Tris acetate buffer	No	Yes	No	PMSF, protease inhibitor cocktail	100,000 g for 3 h	[75]
4B1	Potassium phosphate buffer	Yes	No	No	PMSF, protease inhibitor cocktail	N/A	[76]

N/A = not available; ** Mops = 3-(N-morpholino) propanesulfonic acid.

**Table 3:** Solubilization, purification, and reconstitution of expressed human CYPs from *E. coli* membranes.

CYP	Detergent	Column (s)	OxR	Cytochrome *b_5_*	Specific Content (nmol/mg Protein)	Reference
2E1	n-octylglucoside	S-Sepharose Hydroxyapatite DEAE-Sepharose Hydroxyapatite	N/A	N/A	15.8	[7]
2E1	n-octylglucoside	S-Sepharose	Rabbit liver	N/A	2	[49]
3A4	Sodium cholate	Octylamino-Sepharose Cosmogel DEAE KB Type-S Cosmogel CM Hydroxylapatite	Rabbit liver	Human liver	23	[42]
2E1&2B4	Tergitol NP-10	S-Sepharose (CYP2E1) high-resolution hydroxyapatite (CYP2B4)	N/A	N/A	N/A	[43]
3A4+OxR	Emulgen 911	2′,5′-ADP Sepharose affinity	Fused OxR	N/A	150–200 per L of culture	[67]
1A1	Sodium cholate Triton N-101	DEAE-Sephacel CM-Sepharose fast-flow Hydroxylapatite	Rabbit liver	Human liver	10–15 per L of culture	[44]
2E1	Sodium cholate Triton N-101	DEAE-Sephacel CM-Sepharose fast-flow Hydroxylapatite	Rabbit liver	Human liver	160 per L of culture	[68]
1A2	Sodium cholate Triton N-101	DEAE-Sephacel CM-Sepharose fast-flow	Rabbit liver	Human liver	225–245 per L of culture	[38]
17A1-OxR	Emulgen 911	DE-52 2′,5′-ADP-Sepharose 4B LKB Ultragel AcA34	Co-expressed	N/A	3.8	[69]
3A5	Sodium cholate Triton N-101	DEAE-Sephacel CM-Sepharose fast-flow Hydroxylapatite	Rabbit liver	Human liver	260 per L of culture	[70]
2D6	C_12_E_9_	Ni^2+^-NTA-agarose DEAE-Sephacel HTP hydroxylapatite	Rat liver	N/A	20–40 per L of culture	[77]
2D6	Triton X-114	*E. coli* flavodoxin affinity Biogel HTP hydroxylapatite	Rabbit liver	Human liver	90 per L of culture	[39]
2E1-OxR	n-octylglucoside	S-Sepharose Hydroxyapatite DEAE-Sepharose Hydroxyapatite	Co-expressed	Rabbit liver	0.11	[82]
1A1-OxR	Emulgen 911	DE-52 2′,5′-ADP agarose BioGel HTP hydroxylapatite	Co-expressed	Human liver	25 per L of culture	[71]
27A1	Emulgen 913	DEAE-cellulose Hydroxylapatite	Rat liver	N/A	3.5	[84]
1A2-OxR	Sodium cholate	octyl-Sepharose Hydroxylapatite Adrenodoxin-Sepharose	Co-expressed	N/A	15	[85]
1B1-OxR	Emulgen 911	DE-52 2′,5′-ADP agarose BioGel HTP hydroxylapatite	Co-expressed	Human liver	35 per L of culture	[45]
3A5	Sodium cholate Triton N-101	DEAE-Sephacel CM-Sepharose (fast-flow) Hydroxylapatite	Recombinant	Recombinant	9.2	[72]
2A6	Renex-690	Ni^2+^-NTA agarose Bio-Gel HTP hydroxylapatite	Rabbit liver	N/A	12.35	[46]
2B6	Sodium cholate Triton N-101	DEAE-Sephacel Amberlite XAD-2 CM-Sepharose Hydroxylapatite	*E. coli* expressed rat OxR	N/A	25–80 per L of culture	[47]
2D6-OxR	Sodium cholate Triton N-101	DEAE-Sephacel Hydroxylapatite	Co-expressed	N/A	1–3	[27]
1A2-HDJ-1	Emulgen 911	Mono Q Hi-Trap 2′-5′-ADP-Sepharose Sephacryl S-200 HR 2′-5′-ADP-Sepharose	Expressed from *E. coli*	N/A	60–120 per L of culture	[26]
2B6-GroES/EL	Sodium cholate Triton N-101	TOYOPEARL DEAE-650M TOYOPEARL SP-550C Hydroxyapapite	Commercial purchased	N/A	8.2	[64]
4X1	Sodium CHAPS	Ni^2+^-nitriloacetic acid	Co-expressed	N/A	100–200 per L of culture	[52]
2S1	CHAPS	NTA agarose	Co-expressed	N/A	16	[48]
1A1-OxR	Emulgen 911 Sodium cholate	Ni-NTA agarose	Co-expressed	N/A	N/A	[8]
2C10 &2C9	Emulgen 911 Sodium cholate	DEAE-Sephacel Hydroxylapatite	Rabbit liver	Human liver	5–19 per L of culture	[24]
4A11	CHAPS	Ni-NTA agarose	Co-expressed	N/A	125–320 per L of culture	[74]
2J2	CHAPS	Ni-NTA agarose	Rat liver	N/A	16–18.6 per L of culture	[75]
39A1-GroEL/ES	CHAPS TritonX100	Ni-NTA-protino Ion-exchange Source S	Recombinant	N/A	N/A	[86]
2J2-GroEL/ES	CHAPS	Ni^2+^-NTA agarose	Rat liver	N/A	140–230	[9]

N/A = not available.

## Data Availability

This study is a review article and contains no supporting data; all the literature works were cited within the manuscript from which the details were taken.
